# Arthroscopic management of large subchondral talar cyst: a novel treatment strategy

**DOI:** 10.1093/jscr/rjad276

**Published:** 2023-06-01

**Authors:** Rohan Dahiya, Rajkumar Sundarapandian, Abhinav Nair, Anand Pillai

**Affiliations:** Trauma and Orthopaedics, Wythenshawe Hospital (Manchester University NHS Foundation Trust), Wythenshawe, UK; Trauma and Orthopaedics, Wythenshawe Hospital (Manchester University NHS Foundation Trust), Wythenshawe, UK; Trauma and Orthopaedics, Wythenshawe Hospital (Manchester University NHS Foundation Trust), Wythenshawe, UK; Trauma and Orthopaedics, Wythenshawe Hospital (Manchester University NHS Foundation Trust), Wythenshawe, UK

## Abstract

Subchondroplasty is a novel and minimally invasive technique used to treat large subchondral talar cysts. It avoids soft tissue scarring, the need for osteotomies, bone grafting and its associated complications as seen with conventional approaches. Subchondroplasty involves arthroscopically approaching the cyst and injecting it with calcium phosphate paste injection, which undergoes an endothermic process and crystallizes in a manner that resembles cancellous bone to fill the defect. This case series presents two patients who underwent subchondroplasty: a 44-year-old female nurse with atraumatic right ankle pain of 2 years with a CT scan revealing a large subchondral cyst over the medial talar dome and a 55-year-old male chemical plant worker with left ankle pain following a biking accident 6 years ago whose CT scan too showed a large subchondral cyst in the medial talus. Both tolerated the surgery well, with significant reductions noted in MOXFQ scores at the 1-year follow-up.

## INTRODUCTION

Subchondral talar cysts are osteochondral defects in the talus and are a source of chronic ankle pain [1]. These lesions are challenging to treat because of a large articular surface area, comprising 60% of the total talar body surface area. They are devoid of soft tissue attachment and lack sufficient blood supply, leading to poor healing and complications such as osteonecrosis [2].

Surgical treatment of these lesions depends on the lesion’s stage, location and size. Conventional open surgical techniques carry approach-related morbidity, and arthroscopic techniques are restricted to repairing small lesions (i.e. those <150 mm^2^) [3].

Here, we present a novel arthroscopic technique for managing large subchondral cysts (i.e. those larger than 150 mm^2^) of the talus with calcium phosphate injection leading to good clinical patient outcomes.

## CASE REPORT 1

We reviewed a 46-year-old female patient in the clinic who experienced atraumatic right ankle pain over the past 3 years. The patient worked as a nurse and reported dull pain throughout the day that worsened by the end of her shift, thereby restricting her from functioning efficiently in her demanding job. She did not have a relevant past medical history nor did she use any long-term medications. A clinical examination revealed tenderness along the anteromedial joint line. However, she had a full range of motion and could walk with some discomfort. Her MOXFQ score was 45/80.

**Figure 1:**
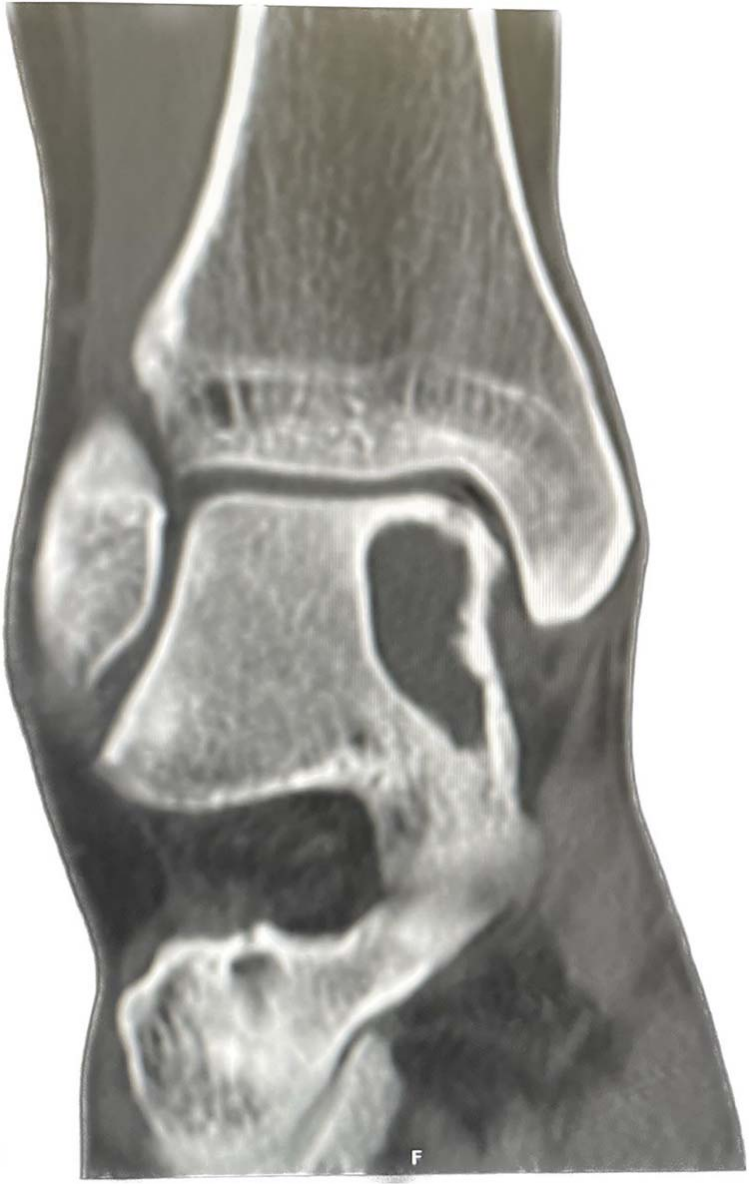
Pre-op Patient 1.

An X-ray of the ankle showed a radiolucency at the anteromedial aspect of the talus, suggesting a subchondral cyst. This lesion measured 18 × 18 × 11 mm on the CT scan in the medial patellar dome with a focal vacuum at the medial talocrural joint. The overlying cortex was thinned out with medial focal cortical defect. There was no collapse of the articular surface (Fig. 1).

**Figure 2:**
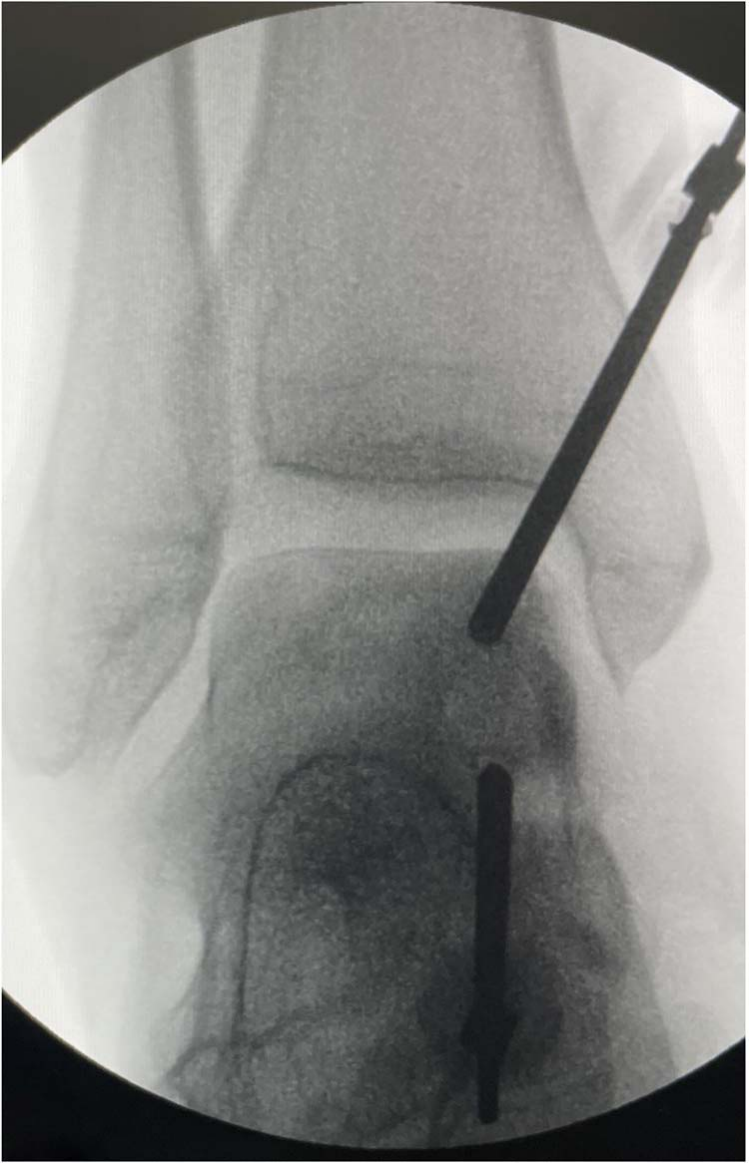
Intra-op.

To address the subchondral cyst, we performed an arthroscopic subchondroplasty using calcium phosphate paste. A full-thickness articular cartilage loss corresponded to the subchondral cyst at the medial talar dome. The subchondral cyst was identified under fluoroscopy, and a trocar was inserted into the cyst under X-ray guidance. Next, 3 cc of calcium phosphate paste was injected into the cyst guided by fluoroscopy to obtain satisfactory filling and ensuring no leakage into the joint space (Fig. 2). This was a day case procedure, and the patient returned home the same day.

Following surgery, the patient was advised not to bear weight on the operated limb for 4 weeks. She was reviewed in the clinic with X-rays 4 weeks after surgery and provided with a walking boot to weight bear for an additional 4 weeks.

**Figure 3:**
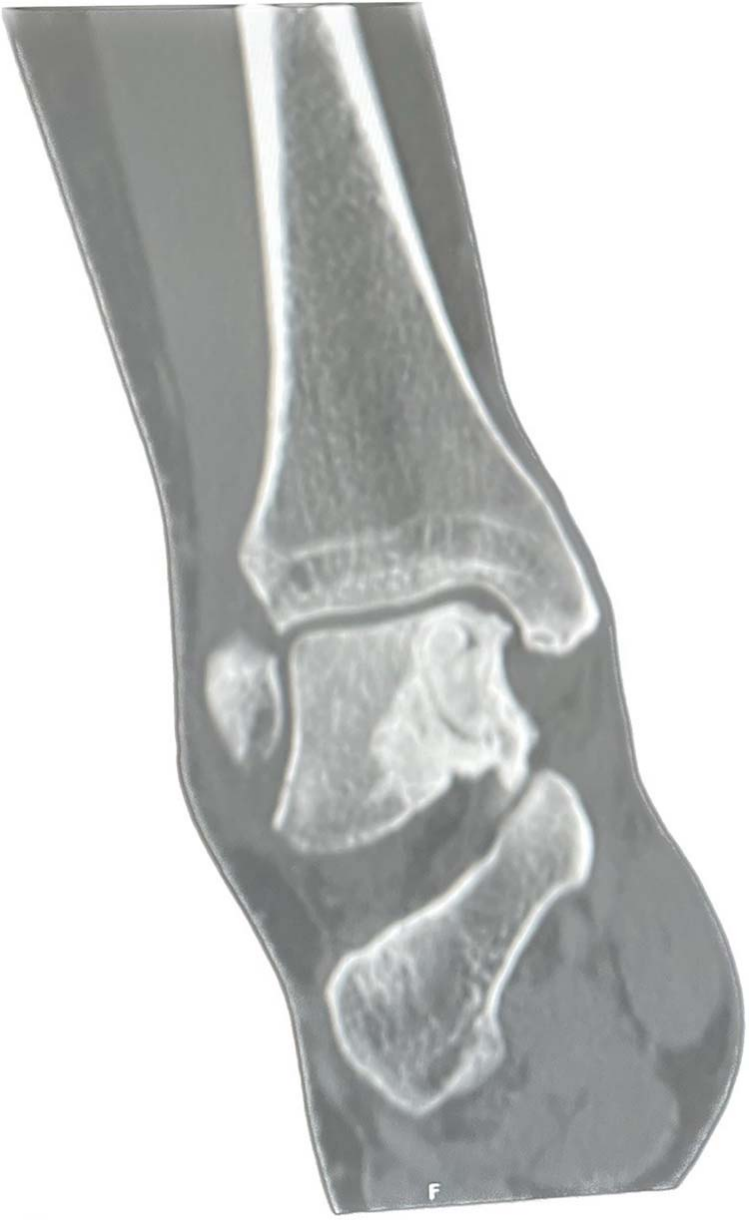
Post-op Patient 1.

A CT scan was performed 6 months later, which showed that more than 50% of the cyst was replaced by bone substitute material and integrated with the surrounding trabecular network (Fig. 3). The patient was pain-free and walked without any discomfort at the 1-year follow-up appointment. The range of motion in her ankle was preserved, and she was able to function better at work. Moreover, her MOXFQ score reduced to 28/80 (Table 1) at the 1-year follow-up.

**Table 1:** MOXFQ score with subchondroplasty.

	**Pre-op**	**1 year post-op**
Patient 1	45/80	28/80
Patient 2	48/80	23/80

## CASE REPORT 2

We also reviewed a 55-year-old male who presented with left ankle pain following a biking accident 6 years prior. Initial imaging showed no bony injury, and he continued to function, albeit with discomfort.

**Figure 4:**
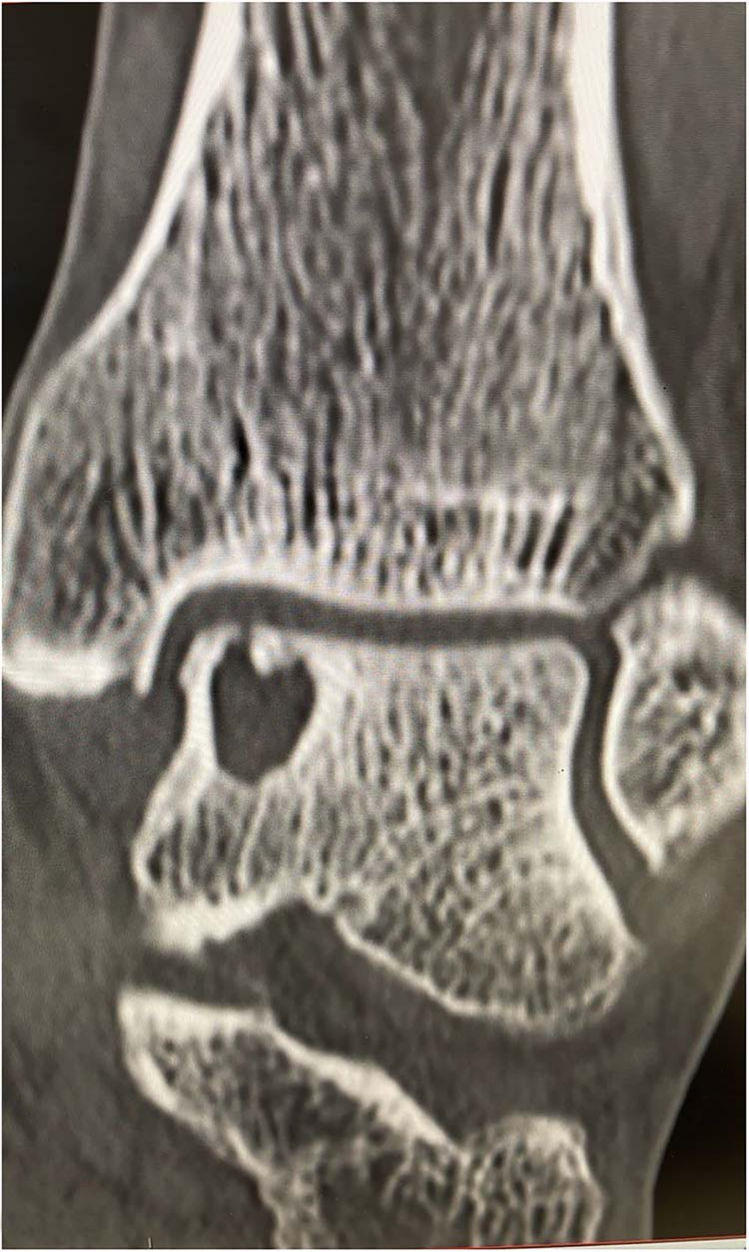
Pre-op Patient 2.

He presented with no relevant medical history and worked as a chemical plant supervisor, which required continuous movement throughout the day. Clinical examination revealed deep-seated pain around the medial aspect of the ankle with an intact range of motion and ankle alignment. His MOXFQ score was 48/80. CT scan revealed a large but well-contained, subchondral cyst in the medial talus of the ankle (Fig. 4).

Subchondroplasty was performed using the technique described above, and similar post-operative precautions were advised.

**Figure 5:**
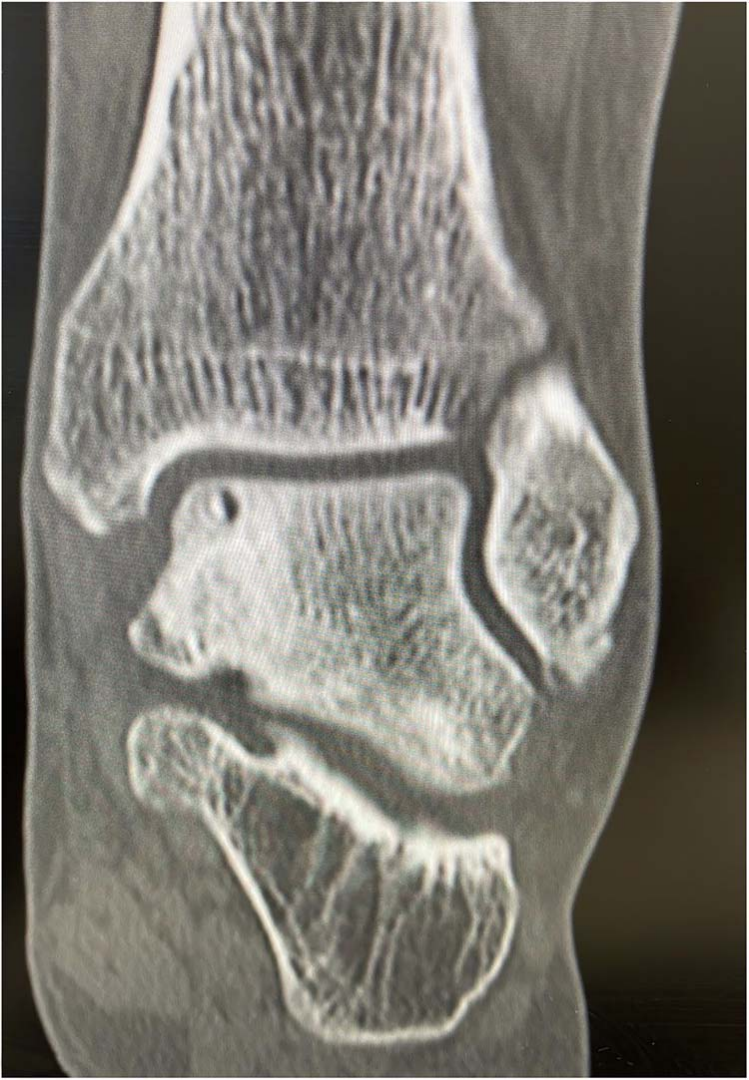
Post-op Patient 2.

The patient was pain-free and comfortable at 6 months following the procedure. CT scan performed at 6 months showed more than 50% of the defect was successfully replaced with bone substitute material and integrated with the trabecular network (Fig. 5). There was a significant reduction in the patient’s MOXFQ score (Table 1) at the 1-year follow-up to 23/80.

## DISCUSSION

The osteochondral cyst of the talus is a common source of pain that can limit function. Treatment options are typically dictated by the site, size and stage of the lesion. Small lesions that measure < 150 mm^2^ are amenable for arthroscopy and microfracture, which stimulates fibrocartilage formation by the marrow elements. Conversely, larger lesions that measure 150 mm^2^ or greater are at risk of articular collapse, and the treatment strategy would involve reinforcing subchondral structural support with a bone graft. These lesions are difficult to treat arthroscopically and require open surgery with a medial or lateral malleolar osteotomy to access the lesion [3]. However, open surgical techniques carry approach-related complications such as infection, risk of neurovascular injury, delayed or non-union of osteotomy site, wound healing complications and implant-related complications. Additionally, bone graft harvest to fill these cysts can cause donor site-related morbidity [3–5].

We used an arthroscopic, minimally invasive technique (detailed in the case series), to treat large subchondral cysts, which avoided the aforementioned complications. Furthermore, patients reported little to no pain post-operatively and returned home on the same day of the procedure.

These outcomes were possible because of the availability of injectable calcium phosphate mineral compound, which crystallizes and hardens quickly in an endothermic reaction at 37 degrees Celsius to form nanocrystalline microporous scaffolds. These compounds are engineered mineral compound bone graft substitutes that mimic cancellous bone properties. They are eventually resorbed and replaced with new bone during the healing process through cell-mediated remodelling [6, 7]. This process reinforces structural support to sub-chondral bone and prevents collapse of the articular surface, thereby preventing early degenerative joint disease [8].

In the study conducted by Sato *et al*., the authors used Calcium phosphate paste to fill a bone defect in a total knee arthroplasty procedure and noted the material’s stiffness as 80 Mpa after 10 days. Their patients could bear weight on the operated knee 10 days after surgery [9]. We advised our patients to be non-weight bearing for 4 weeks after the procedure. We injected calcium phosphate paste into the cyst to provide structural support to the articular surface and to prevent collapse. We wanted to ensure adequate integration of the bone graft substitute before commencing weight bearing. We did not have literature evidence to guide us regarding the optimal time to commence weightbearing and thus chose a safer option of keeping them non-weightbearing for 4 weeks.

Our patients reported complete resolution of symptoms following the procedure with significant reduction in MOXFQ scores. We observed no evidence of avascular necrosis in the most recent imaging of either patient. Similarly, Rona W. Law *et al*. [10] recorded one of the largest case series of subchondroplasty with 28 patients, and they observed no evidence of avascular necrosis in any patient 1 year after the procedure. Additionally, 21 patients in their series demonstrated complete resolution of reported pre-operative symptoms.

## CONCLUSION

This study presented a novel arthroscopic treatment strategy for large subchondral talar cysts in a case series. This approach is minimally invasive and guided by fluoroscopy to fill the sub-chondral cyst with calcium phosphate paste to confer structural support and prevent articular surface collapse. Our patients reported complete resolution of all symptoms at a recent clinic follow-up.

## CONFLICT OF INTEREST STATEMENT

None declared.

## DATA AVAILABILITY STATEMENT

The data that support the findings of this case report are available upon reasonable request to the corresponding author. All required links or identifiers for the data are present in the manuscript as described. Any requests for data should be directed to the corresponding author at rohan7@doctors.net.uk.
